# The prevalence of hypoxemia among pediatric and adult patients presenting to healthcare facilities in low- and middle-income countries: protocol for a systematic review and meta-analysis

**DOI:** 10.1186/s13643-020-01326-5

**Published:** 2020-03-30

**Authors:** Felix Lam, Rami Subhi, Jason Houdek, Kate Schroder, Audrey Battu, Hamish Graham

**Affiliations:** 1 Clinton Health Access Initiative, Inc., 383 Dorchester Ave, Suite 400, Boston, MA 02127 USA; 2 Centre for International Child Health, University of Melbourne, MCRI, Royal Children’s Hospital, Level 2 East, 50 Flemington Road, Parkville, Victoria 3052 Australia

**Keywords:** Systematic review, Meta-analysis, Hypoxemia, Pulse oximetry

## Abstract

**Background:**

Hypoxemia is a severe condition associated with high rates of mortality, particularly in low- and middle-income countries (LMICs) with poor access to oxygen therapy. Despite its clinical significance, there have been few studies to describe the burden of hypoxemia. Thus, the primary objective of this study is to systematically describe the prevalence of hypoxemia among pediatric and adult patients in low- and middle-income countries.

**Methods/design:**

Standard systematic review methods will be used. Bibliographic databases (MEDLINE, EMBASE, CINAHL) will be searched from 1998 onwards. The search strategy aims to identify studies that have measured peripheral blood oxygen saturation (SpO_2_) in children and adults presenting to health facilities in LMICs. Studies will be included if oxygen saturation measurements by pulse oximetry were measured. No studies will be excluded based on study design though patients recruited from intensive care units and post-operative care will be excluded. The primary outcome is the prevalence of hypoxemia on presentation to the healthcare facility. We define hypoxemia on the basis of SpO_2_ measurements, and use a threshold of SpO_2_ less than 90% at sea level though allow for a lower threshold for studies conducted at higher altitude and where justified. Standardized tools will be used to extract data on number of patients with SpO_2_ measurements, number of patients with hypoxemia, patient population characteristics, and study characteristics. Quality of the included studies will be assessed using the “Checklist for Prevalence Studies” developed by the Joanna Briggs Institute. If there are enough studies to do so, we will conduct meta-analysis using a random effects model to estimate prevalence of hypoxemia and conduct subgroup analyses by age and disease groups.

**Discussion:**

Hypoxemia is a critical condition and understanding the burden of hypoxemia may support decision-making in LMICs to deploy pulse oximeters and oxygen treatments more efficiently to address diseases and patient populations with the highest burden. Previous studies on hypoxemia prevalence have focused too narrowly on a few diseases or specific patient populations (e.g., pneumonia in children under five) whereas any effort to improve access to oxygen requires understanding of the potential demand for oxygen for all diseases and population groups. Governments, UN agencies, donors, and NGOs are investing strongly to improve oxygen systems in LMICs. Effective oxygen system planning requires estimation of oxygen need, informed by robust data on hypoxemia prevalence and admission patterns at all the levels of the health system. This study aims to fill that gap by providing comprehensive estimates of hypoxemia prevalence.

**Systematic review registration:**

PROSPERO CRD42019136622.

## Background

Hypoxemia is a severe condition characterized as insufficient oxygen in the blood. Many diseases can cause hypoxemia, including pneumonia, malaria, neonatal sepsis, anemia, pulmonary diseases in adults, and adult heart failure, and can cause hypoxemia in different ways. For pneumonia, (one of the most common causes of hypoxemia especially in children), hypoxemia is primarily caused by obstruction of oxygen exchange in the alveoli of the lungs. A systematic review found that hypoxemia is a strong predictor of mortality for pneumonia in children, and other studies have also found hypoxemia to be associated with mortality and other poor outcomes for other diseases [1–6]. Patients in low- and middle-income countries (LMICs) are disproportionately affected as oxygen therapy, an effective treatment of hypoxemia, is not widely accessible [7, 8]. Despite the clinical significance of hypoxemia, there are few studies that have estimated the burden of hypoxemia. We found one systematic review of the prevalence of hypoxemia [9]. The systematic review included 24 studies and found hypoxemia prevalence estimates ranging between 2-23% across eight conditions in children. However, the review was limited to children younger than 12 years, and as oxygen is applicable to adolescents and adults with hypoxemia as well, an updated review including older age groups would help inform initiatives trying to improve oxygen access in LMICs. Furthermore, prevalence of diseases across LMICs is likely to differ and so providing rigorous estimates of hypoxemia prevalence across a wider range of diseases than that included in the earlier systematic review will also help improve plans for increasing oxygen access.

## Methods/design

### Aims and objectives

The aim of the study is to describe the prevalence of hypoxemia among pediatric and adult patients presenting to healthcare facilities in LMICs.

### Selection criteria

The search strategy and selection criteria are described below.

#### Participants

Patients of all ages presenting to a health facility with acute illnesses for whom oxygen saturation measurements by pulse oximetry (SpO_2_) were measured. The following is a comprehensive, though non-exhaustive list of conditions for which studies may report hypoxemia prevalence:

- Respiratory conditions:
  - Pneumonia/bronchiolitis/acute lower respiratory infection (ALRI)
  - Tuberculosis
  - Neonatal—prematurity/respiratory distress syndrome (RDS)
  - Chronic obstructed pulmonary disease (COPD)
  - Asthma
  - HIV-related infections—pneumocystis pneumonia (PJP)
- Non-respiratory conditions:
  - Infections: sepsis, meningitis/encephalitis, malaria
  - Neurological: neonatal encephalopathy/asphyxia, stroke, seizures
  - Malignancies
  - Trauma/injury
  - Pregnancy—post-partum hemorrhage (PPH), eclampsia, and pre-eclampsia
  - Malnutrition (broad but will yield relevant studies): severe acute malnutrition (SAM)/moderate acute malnutrition (MAM)

The study will exclude studies of patients recruited from intensive care units (likely multi-factorial etiology of hypoxemia and mechanical ventilation) and post-operative care, or who are admitted for elective procedures. We will also exclude studies at high risk of selection bias based on quality assessments conducted by the reviewers (See the “Quality assessment” section).

#### Intervention

Only studies in which pulse oximetry was conducted will be included in the review. Pulse oximetry uses the differences in absorbance of two different wavelengths of light by oxygenated and deoxygenated hemoglobin to non-invasively estimate arterial oxygen saturation (recording peripheral oxygen saturation, or SpO_2_). It is well correlated with arterial oxygen saturation measured by blood gas analysis (PaO_2_), and is much cheaper and does not require the infrastructure and upkeep of blood gas machines, making the technology more applicable to LMIC settings. The World Health Organization recommends using pulse oximetry for detection of hypoxemia [10].

#### Setting

Studies conducted in low and lower-middle income countries as defined by the World Bank 2019 country classification list will be included in the study [11]. We will include only studies conducted in a health facility (any level) though we will exclude studies conducted within intensive care units and post-operative care.

#### Timing

Pulse oximetry is a relatively recent technology in LMICs. Several studies highlighting poor access to it in the perioperative setting have been published in the mid-2000s, and it was part of WHO’s Surgical Safety Checklist in 2009. To ensure a sensitive search strategy, we have included studies published a decade earlier, since January 1, 1998.

#### Study designs

No studies will be excluded based on the study design. There are few studies designed specifically to estimate hypoxemia prevalence as the primary outcome, but rather, hypoxemia is included as part of the clinical studies for a variety of other reasons (e.g., control variable or identification of severe illness). Thus, we will take a broad review of all studies that measured peripheral blood oxygen using pulse oximetry.

#### Outcomes

The primary outcome of interest is the prevalence of hypoxemia on presentation to the healthcare facility. We define hypoxemia on the basis of SpO_2_ measurements, and use a threshold of SpO_2_ less than 90% at sea level. With higher altitude, the partial pressure of oxygen reduces, and studies have demonstrated lower levels of what is considered normal SpO_2_ in healthy populations. Therefore, we will allow for adjustment in the definition of hypoxemia with altitude, where authors took this into account, and using an alternate definition with a lower threshold of SpO_2_. For studies that applied a threshold of hypoxemia likely to overestimate prevalence (such as a threshold level above SpO_2_ of 90% at sea level), we will exclude the study unless the authors can be contacted to provide prevalence data for the standard threshold or we are able to obtain the dataset for re-analysis to the standard threshold definition. We will include all studies which collected SpO_2_ measurements, even if hypoxemia was not the primary outcome.

#### Language

Studies published in English, French, and Spanish will be included in the review.

### Information sources

We will search for published studies and datasets using the following databases: MEDLINE, PubMed (for studies published, studies in the past year that have not yet been indexed on MEDLINE), EMBASE, CINAHL, and the Index Medicus for each WHO region, and Google Scholar (the first 500 abstracts will be reviewed).

To ensure comprehensiveness of the literature search, we will also scan the reference lists of included studies or relevant reviews identified. We will also seek out unpublished data sets from key researchers in the field. We will contact researchers who are known to have done studies in low- and middle-income countries where oximetry was recorded.

### Search strategy

The search strategy aims to identify studies that have collected SpO_2_ measurements using pulse oximetry on patients meeting the criteria described above. The specific search strategies will be developed in consultation with a health sciences research librarian with expertise in systematic reviews. A draft MEDLINE search strategy is included in Additional file 1: Appendix 1 and has been informed by prior experience in conducting a systematic review on hypoxemia prevalence in children less than 12 years [9]. After the MEDLINE search strategy is finalized, it will be adapted to the syntax and subject headings of other databases.

### Study records

#### Data management

Search results will be documented in an electronic database such as Excel or Google Sheets. A template for documenting search results is included in Additional file 1: Appendix 2. A standardized form (Additional file 1: Appendix 3) will be used to screen and select studies. Each publication will be assigned a “Report ID,” and where there are multiple publications coming from the same study, each study will also be assigned a single “Study ID.” During the screening process, we will identify duplication of studies reported across multiple publications and multiple databases and remove duplicates from our study database. For studies meeting the screening criteria, the form will also be used to extract relevant study variables for analysis and quality assessment. We will pilot the study forms using the first 20 studies found using the search strategy and amend the form as necessary before implementing it on the entire search results. The full text articles of studies selected for inclusion will be uploaded to Endnote.

#### Selection process

Two independent reviewers will screen the full text of all studies resulting from the search strategy and use the data extraction and assessment form to screen studies for inclusion. Disagreements between the two reviewers will be screened by a third independent reviewer. For studies where any of the eligibility criteria are unclear, the reviewers will attempt to contact the study’s corresponding author for clarification. Three attempts will be made to contact the study’s corresponding author. If all three attempts fail, the study will be excluded from the review.

#### Data collection process

For all studies meeting the inclusion criteria, one reviewer will extract data from the reports. The data extraction and assessment form will be used, and the data will be entered into an electronic database. For studies not reporting summary statistics on SpO_2_, the reviewer will reach out to the study’s corresponding author to request for the study’s dataset. We will use the draft letter in Additional file 1: Appendix 4. Three attempts will be made to contact the study’s corresponding author. If all three attempts fail, the study will be excluded from the review.

### Data items

The data items we aim to extract include the following:

- Numbers of patients with hypoxemia at presentation or first recording thereafter and total number of patients screened
- Patient population information, including number of patients included in the study, age range of study population, inclusion and exclusion criteria, and diagnostic case definitions (e.g., WHO defined severe pneumonia)
- Study characteristics, including the study’s aim, the study design, the healthcare setting where the study was conducted, elevation above sea level where study was conducted, and time period where the study was conducted

### Outcomes and prioritization

The primary outcome of interest is the proportion of patients with hypoxemia (SpO_2_ < 90%) on presentation to the facility. If the primary outcome is not reported, a secondary outcome using another SpO_2_ cutoff may be used if adequate justification was used for the alternate cutoff. We will contact authors for the dataset and re-analyze the data to standardize the definition of hypoxemia if possible.

### Quality assessment

We will use the “Checklist for Prevalence Studies” developed by the Joanna Briggs Institute for assessing study quality at the study level [12]. The ‘Checklist for Prevalence Studies’ assesses study quality along nine components: appropriate sampling frame, appropriate sampling design, adequate sample size, detailed description of study subjects and setting, sufficient coverage of sample, valid methods for identifying the condition, standard and reliable measurement of the condition, appropriate statistical analysis, and adequate response rate. Study quality will be assessed by two reviewers, and with agreement of the two reviewers, we will exclude studies where there was high potential for selection bias, such as where there was selective recruitment of sub-populations of patients with more severe illness, or where oximetry was not measured and recorded in a systematic way within the study. Where there are disagreements between the two reviewers, a third independent reviewer will assess the study quality for a final determination.

We will also assess for publication bias of selective populations by seeking out unpublished data from researchers. Published studies may represent populations with high prevalence of hypoxemia, such as cohorts in large tertiary hospitals wherein severity of illness is usually higher.

Some studies may also have selective definitions of hypoxemia that may result in over- or under-reporting of hypoxemia. Where possible, we will request the data from the authors for re-analysis to standardize the definition of hypoxemia to SpO2 < 90%. As mentioned above, we will allow for lower thresholds of hypoxemia due to altitude where authors have provided such information.

We will also assess for the possible presence of small sample bias in the publication literature using funnel plots and test for asymmetry using Egger’s test [13].

### Data synthesis

Statistical analysis will be done using the Stata 16 statistical software (Statacorp, College Station, Texas, USA). We will report hypoxemia prevalence for the following subgroups: age, diagnosis (and severity), level of health facility (primary, secondary, tertiary), and altitude. For age, we will stratify results into three age categories: neonates (< 28 days of age), infant/young child (1-59 months), older child (5-17 years), and adults (> 18 years). We will stratify disease based on clinical diagnosis and severity using standardized case definitions where these exist. The clinical criteria for defining diseases are likely to vary across studies. For example, pneumonia in some studies may be defined by chest radiography while in other studies pneumonia may be defined by tachypnea alone. We will compare reported case definitions to standardized global guidelines (e.g., WHO guidelines) and combine data from studies using comparable case definitions as much as possible. We will classify altitude as high (≥ 1500 m above sea level) and low altitude (< 1500 m above sea level).

We will use a random-effects meta-analysis to obtain a point prevalence of hypoxemia (95% confidence interval), pooling together all studies, by age, clinical diagnosis and severity, health facility type, and high/low altitude. The calculated point prevalence of hypoxemia will represent an average of distributions rather an estimate of a one true prevalence. We expect a moderate-high degree of heterogeneity between studies. Depending on the number of available studies, we will attempt to reconcile some of these differences by calculating point prevalence for more targeted subgroups—e.g., prevalence of hypoxemia in 1-59 month old children for severe pneumonia in secondary or tertiary facilities. We will determine the *I*^2^ value (index of the proportion of total variation across studies that is due to heterogeneity rather than to chance) for each estimate of prevalence as an indication of the between-study heterogeneity. If the point-prevalence by the described analysis is likely to be misleading due to irreconcilable heterogeneity between included studies, we will present these results as the medians and inter-quartile ranges of the proportion of hypoxemic children in the studies within each subgroup.

## Discussion

Hypoxemia is a critical condition and understanding the burden of hypoxemia may support decision-making in LMICs to deploy pulse oximeters and oxygen treatments more efficiently to address diseases and patient populations with the highest burden. Previous studies on hypoxemia prevalence have focused too narrowly on a few diseases or specific patient populations (e.g., pneumonia in children under five) whereas any effort to improve access to oxygen requires understanding of the potential demand for oxygen for all diseases and population groups. This study aims to fill that gap by providing comprehensive estimates of hypoxemia prevalence. Furthermore, governments, UN agencies, donors, and NGOs are investing strongly to improve oxygen systems in LMICs. Effective oxygen system planning requires estimation of oxygen need, informed by robust data on hypoxemia prevalence and admission patterns at all the levels of the health system. Currently, the lack of quality data on hypoxemia prevalence is compromising efforts to predict oxygen needs and efficiently plan scale up of pulse oximetry and oxygen systems.

Limitations of this study are that pulse oximetry is not widely used in low-resource settings and while it is used in clinical research, pulse oximetry results are often not reported as a primary outcome of the study. We aim to mitigate this limitation by contacting study authors to obtain data for re-analysis. A key logistical factor in the study’s success will be the cooperation of those authors.

## Supplementary information

**Additional file 1: Appendices 1-5**

**Additional file 2: PRISMA-P checklist**

## Data Availability

Not applicable

## Abbreviations

ALRI: Acute lower respiratory infection
COPD: Chronic obstructive pulmonary disorder
HIV: Human immunodeficiency virus
LMIC: Low- and middle-income countries
MAM: Moderate acute malnutrition
PaO_2_: Partial pressure of arterial oxygen
PJP: Pneumocystis pneumonia
PPH: Post-partum hemorrhage
RDS: Respiratory distress syndrome
SAM: Severe acute malnutrition
SpO_2_: Peripheral oxygen saturation
WHO: World Health Organization
