# Supplementary material for: The prevalence of hypoxemia among pediatric and adult patients presenting to healthcare facilities in low- and middle-income countries: protocol for a systematic review and meta-analysis
Source: Syst Rev. 2020 Mar 30;9:67. doi: 10.1186/s13643-020-01326-5 (PMC7106676; doi:10.1186/s13643-020-01326-5)
Supplement: Supplementary file 1 — Additional file 1: Appendices 1-5 [file 13643_2020_1326_MOESM1_ESM.docx]

# Additional file: Appendices 1-5

# Appendix 1: Draft MEDLINE search strategy

The draft MEDLINE search strategy uses a combination of the following search terms and returned 2,045 results:

- Terms for hypoxia/oximetry AND list of diseases/conditions AND LMIC filter, (search 34 below), OR
- Terms for hypoxia/oximetry AND risk factors AND LMIC filter (search 35)
- Terms for hypoxia/oximetry AND terms for predictors/point of care tests AND LMIC filter (search 36), OR
- Terms for hypoxia/oximetry AND incidence/prevalence (search 37), OR
- Terms for hypoxia AND terms for oximetry/oxygen AND LMIC filter  (search 38)

The specific search strategy is as follows (with the number of studies returned indicated in parentheses at the end of the search terms):

1     hypoxia/ (61575)

2     hypoxia/di (1974)

3     (anox?emia or hypox?emia).tw,kf. (18208)

4     exp Respiratory Tract Infections/cl, co, mo (57629)

5     pneumonia/cl, co, mo or bronchopneumonia/cl, co, mo or pleuropneumonia/cl, co, mo or exp pneumonia, bacterial/cl, co, mo or pneumocystis/cl or pneumonia, viral/cl, co, mo (14167)

6     exp Malaria/cl, co, mo [Classification, Complications, Mortality] (6702)

7     exp Tuberculosis/cl, co, mo [Classification, Complications, Mortality] (28190)

8     exp Pulmonary Disease, Chronic Obstructive/cl, co, mo [Classification, Complications, Mortality] (11013)

9     exp Asthma/cl, co, mo [Classification, Complications, Mortality] (13055)

10     exp Sepsis/cl, co, mo [Classification, Complications, Mortality] (23902)

11     exp Meningitis/cl, co, mo [Classification, Complications, Mortality] (9794)

12     exp Brain Diseases/cl, co, mo (225360)

13     exp Multiple Trauma/cl, co, mo [Classification, Complications, Mortality] (3875)

14     exp Pregnancy Complications/cl, co, mo (24104)

15     exp neoplasms/cl, co, mo (482486)

16     exp Oximetry/ (14374)

17     Oxygen/ad, bl, tu, th [Administration & Dosage, Blood, Therapeutic Use, Therapy] (60957)

18     (oximetry or oxygen-saturation).tw,kf. (31019)

19     nutrition disorders/cl, co, mo or malnutrition/cl, co, mo or exp severe acute malnutrition/co, mo or starvation/cl, co, mo (8211)

20     risk factors/ (760084)

21     "reproducibility of results"/ or "sensitivity and specificity"/ or "predictive value of tests"/ or roc curve/ or Mobile Applications/ or exp Point-of-Care Systems/ (777493)

22     incidence/ or prevalence/ (483978)

23     developing countries/ (71887)

24     (austere or (limited adj2 resource*) or (low adj2 resource*) or (transitioning adj econom*) or (third adj world) or LMIC or LMICs or (lami adj countr*) or (transitional adj countr*) or (low adj gdp) or (low adj gnp) or (low adj gross adj domestic) or (low adj gross adj national) or ((emerging or developing or (low adj income) or (middle adj income) or (low adj3 middle) or underdeveloped or under-developed or (less* adj developed) or underserved or under-served or deprived or poor*) and (countr* or nation*1 or econom* or population or world))).tw,kf. (339633)

25     exp africa/ (245588)

26     americas/ or exp caribbean region/ or exp central america/ or latin america/ or mexico/ or exp south america/ (232600)

27     europe/ or exp europe, eastern/ or exp transcaucasia/ (268933)

28     antarctic regions/ or exp atlantic islands/ or exp indian ocean islands/ or exp pacific islands/ (73867)

29     New Guinea/ (2027)

30     asia/ or exp asia, central/ or asia, southeastern/ or borneo/ or cambodia/ or east timor/ or indonesia/ or laos/ or malaysia/ or mekong valley/ or myanmar/ or philippines/ or thailand/ or vietnam/ or asia, western/ or bangladesh/ or bhutan/ or india/ or middle east/ or afghanistan/ or iran/ or iraq/ or jordan/ or lebanon/ or oman/ or saudi arabia/ or syria/ or turkey/ or yemen/ or nepal/ or pakistan/ or sri lanka/ or far east/ or china/ or tibet/ or exp korea/ or mongolia/ (507004)

31     (Afghanistan or Albania or Algeria or Angola or Antigua or Argentina or Armenia* or Aruba or Azerbaijan or Bahrain or Bangladesh or Barbados or Barbuda or Belarus or Byelarus* or Byelorussian or Belorussian or Belorus* or Belize or Benin or Bhutan or Bolivia or Bosnia or Botswana or Brasil or Brazil or Bulgaria or (Burkina adj Fas*) or (Upper adj Volta) or Burma or Burundi or Cambodia or Khmer or Kampuchea or Cameron* or Cameroon* or (Cape adj Verde) or (Cabo adj Verde) or (Central adj African adj Republic) or Chad or Chile or China or Colombia or Comoros or (Comoro adj Island*) or Comores or Mayotte or Congo or Kongo or (Cook adj Island*) or (Costa adj Rica) or (Cote adj D'ivoire) or Croatia or Cuba or Cyprus or (Czech adj Republic) or Czechoslovakia or Djibouti or Dominica or Dominican or (East adj Timor) or (East adj Timur) or Ecuador or Egypt or El-Salvador or (Equatorial adj Guinea) or Eritrea or Estonia or Ethiopia or Fiji or (French adj Somaliland) or Futuna or Gabon or (Gabonese adj Republic) or Gambia or Gaza or (Georgia* adj Republic) or Ghana or Grenada or Guam or Guatemala or Guinea or Guiana or Guyana or Haiti or Herzeg* or Hercegovina or Honduras or Hungary or India or Indonesia or Iran or Iraq or (Ivory adj Coast) or Jamaica or Jordan or Kazakh* or Kenya or Kiribati or Korea or Kosovo or (Kyrgyz adj Republic) or Kyrgyzstan or Kirghizia or Kirghiz or Kirgizstan or Laos or (Lao* adj2 Democratic adj Republic) or (Lao* adj PDR) or Latvia or Lebanon or Lesotho or Basutoland or Liberia or Libya or Lithuania or Macedonia or Madagascar or (Magalasy adj Republic) or Malawi or Malay* or Sabah or Sarawak or Maldives or Mali or (Marshall adj Island*) or Mauritania or Mauritius or (Agalega adj Island*) or Mexico or Micronesia or Moldov* or Mongolia or Montserrat or Montenegro or Morocco or Ifni or Mozambique or Myanma* or Namibia or Nauru or Nepal or (Netherlands adj Antilles) or (Dutch adj Antilles) or (New adj Guinea) or (New adj Caledonia) or Nicaragua or Niue or Niger or Nigeria or (Northern adj Mariana adj Island*) or Nyasaland or Oman or Pakistan or Palau or Panama or (Papua adj New adj Guinea) or PNG or Palestine or Paraguay or Peru or Philipines or Philippines or Phillipines or Phillippines or Poland or (Puerto adj Rico) or Yemen or Romania or Roumania or Rumania or Russia* or Rwanda or Ruanda or (Saint adj Kitts) or (St adj Kitts) or Nevis or (Saint adj Vincent) or (St adj Vincent) or Grenadines or Samoa* or (Navigator adj Island*) or (Saint adj Lucia) or (St adj Lucia) or (Saint adj Helena) or (St adj Helena) or (Sao adj Tome) or (Saudi adj Arabia) or Senegal or Serbia or Seychelles or (Sierra adj Leone) or Slovenia or Slovak* or (South adj Africa) or (Solomon adj Island*) or Somalia or (Sri adj Lanka) or Ceylon or Sudan or Surinam* or Swaziland or Syria or Tajikistan or Tadzhikistan or Tadjikistan or Tadzhik or Tanzania or Thailand or Tibet or Timor-Leste or Togo or (Togolese adj Republic) or Tokelau or Tonga or Trinidad or Tobago or Tunisia or Turkey or Turkmenistan or Turkmen or Tuvalu or Uganda or Ukraine or Uruguay or Urundi or USSR or (Soviet adj Union) or "Union of Soviet Socialist Republics" or Uzbekistan or Vanuatu or (New adj Hebrides) or Venezuela or Vietnam or (Viet adj Nam) or (Wallis adj Futuna) or (United adj Arab adj Republic) or (West adj Bank) or (West adj Indies) or Yemen or Yugoslavia or Zaire or Zambia or Zimbabwe or Rhodesia).tw,kf. (1118756)

32     (africa or americas or caribbean or (central adj America) or (latin adj America) or (south adj America) or (eastern adj Europe) or Transcaucasia or antarctic or (atlantic adj island*) or (indian adj ocean adj island*) or (pacific adj island*) or polynesia or (central adj asia) or (southeast* adj asia) or (south-east* adj asia) or borneo or mekong or (western adj asia) or (middle adj east) or (far adj east)).tw,kf. (206464)

33     23 or 24 or 25 or 26 or 27 or 28 or 29 or 30 or 31 or 32 (2017801)

34     (1 or 3 or 16 or 17 or 18) and (4 or 5 or 6 or 7 or 8 or 9 or 10 or 11 or 12 or 13 or 14 or 15 or 19) and 33 (334)

35     (1 or 3 or 16 or 17 or 18) and 20 and 33 (365)

36     (2 or exp *Respiratory Tract Infections/cl, co, mo or (*pneumonia/cl, co, mo or *bronchopneumonia/cl, co, mo or *pleuropneumonia/cl, co, mo or exp *pneumonia, bacterial/cl, co, mo or *pneumocystis/cl or *pneumonia, viral/cl, co, mo) or exp *Malaria/cl, co, mo or exp *Tuberculosis/cl, co, mo or exp *Pulmonary Disease, Chronic Obstructive/cl, co, mo or exp *Asthma/cl, co, mo or exp *Sepsis/cl, co, mo or exp *Meningitis/cl, co, mo or exp *Brain Diseases/cl, co, mo or exp *Multiple Trauma/cl, co, mo or exp *Pregnancy Complications/cl, co, mo or exp *neoplasms/cl, co, mo or (*nutrition disorders/cl, co, mo or *malnutrition/cl, co, mo or exp *severe acute malnutrition/co, mo or *starvation/cl, co, mo)) and 21 and 33 (1201)

37     *hypoxia/ and 22 (232)

38     (1 or 3) and (16 or 17 or 18 or 21) and 33 (502)

39     34 or 35 or 36 or 37 or 38 (2364)

40     exp animals/ not human*.sh. (4559589)

41     39 not 40 (2278)

42     limit 41 to yr="1998 -Current" (2045)

# Appendix 2: Template for documenting search results

| Search date | Search database | Search terms | Results found |
| --- | --- | --- | --- |
|  |  |  |  |
|  |  |  |  |

# Appendix 3: Data extraction and assessment form

# General Information

| Study ID (surname of first author and year first full report of study was published e.g. Smith 2001) |  |
| --- | --- |
| Report ID |  |
| Report ID of other reports of this study |  |
| Date form completed (dd/mm/yyyy) |  |
| Name/ID of person reviewing/extracting data |  |
| Reference citation |  |
| Study author contact details |  |
| Publication type (e.g. full report, abstract, letter) |  |
| Notes: | |

# Study eligibility

| Study Characteristics | Eligibility criteria  *(Insert inclusion criteria for each characteristic as defined in the Protocol)* | | Eligibility criteria met? | | | Location in text or source *(pg & ¶/fig/table/other)* |
| --- | --- | --- | --- | --- | --- | --- |
|  |  |  | Yes | No | Unclear |  |
| Setting | Low and lower-middle income country | |  |  |  |  |
|  | Study conducted in healthcare facility, not including intensive care unit or post-operative care | |  |  |  |  |
| Participants | Meets selection criteria | |  |  |  |  |
| Types of intervention | Pulse oximetry conducted | |  |  |  |  |
| INCLUDE | | EXCLUDE | | | | |
| Reason for exclusion |  | | | | | |
| Notes: | | | | | | |

**DO NOT PROCEED IF STUDY EXCLUDED FROM REVIEW**

# Characteristics of included studies

## Methods

|  | **Descriptions as stated in report/paper** | **Location in text or source** *(pg & ¶/fig/table/other)* |
| --- | --- | --- |
| **Aim of study** *(e.g. efficacy, equivalence, pragmatic)* |  |  |
| **Design***(e.g. parallel, crossover, non-RCT)* |  |  |
| **Healthcare setting** *(e.g. tertiary hospital, primary care)* |  |  |
| **Facility size** |  |  |
| **Geographic setting**  *(e.g. urban, rural)* |  |  |
| **Elevation above sea level** |  |  |
| **Start date** |  |  |
| **End date** |  |  |
| **Notes:** | | |

## Participants

|  | Description  *Include comparative information for each study group if available* | Location in text or source *(pg & ¶/fig/table/other)* |
| --- | --- | --- |
| Population description  *(from which study participants are drawn)* |  |  |
| Inclusion criteria |  |  |
| Exclusion criteria |  |  |
| Total no. recruited |  |  |
| Age |  |  |
| Notes: | | |

## Outcomes

|  | Description as stated in report/paper | | Location in text or source *(pg & ¶/fig/table/other)* |
| --- | --- | --- | --- |
| Outcome name | Hypoxemia | |  |
| Outcome definition |  | |  |
| Information on pulse oximeter  *(e.g. make/model)* |  | |  |
| Time points measured |  | |  |
| No. of participants measured |  | |  |
| No. of missing participants |  | |  |
| No. with hypoxemia |  | |  |
| Reanalysis required? *(specify)* | Yes No Unclear |  |  |
| Reanalysis possible? | Yes No Unclear |  |  |
| Reanalysed results | Proportion | SE |  |
|  |  |  |  |
| Notes: | | | |

# Appendix 4: Draft letter to study authors

Dear…

We are also investigating the prevalence of hypoxaemia in pediatric and adult patients presenting to health facilities in low and middle income countries. As part of collecting data to support this, we are exploring studies which recorded, as part of the methodology, measurements of oxygen saturations (SpO_2_).

Do you have a data-set representing SpO_2_ levels of infants, children, or adults presenting to health facilities with *any* diagnosis? To be informative these should be data on consecutive patients over a defined period of time, not selected for any particular disease entity or disease severity.

Obtaining an insight into the burden of hypoxaemia in LMICs will help inform the need for oxygen detection and therapy in improving clinical outcomes in LMICs.

If oximetry was *not* done on consecutive patients we would need to know what criteria needed to be met for oximetry to be performed, as well as the demographics of the patients for whom oxygen saturation was measured, and whether these were representative of the study sample and the facility population in general.

We realize that this task may be time-consuming, and so we are willing to sort through de-identified data to extract the relevant information, and perform the appropriate analysis. No further action will be taken using the data without your prior consent.

Your assistance would be much appreciated.

# Appendix 5: Model summary table

| **Group** | **Prevalence of hypoxemia (95% CI)** | **Number of observations** | **Number of studies** |
| --- | --- | --- | --- |
| Neonates |  |  |  |
| - All conditions |  |  |  |
| - Prematurity / RDS |  |  |  |
| - Infections (e.g. sepsis) |  |  |  |
| - Neurological conditions (e.g. HIE, asphyxia) |  |  |  |
| Pediatrics |  |  |  |
| - All conditions |  |  |  |
| - Pneumonia / Bronchiolitis / ALRI |  |  |  |
| - Asthma |  |  |  |
| - HIV-related infections |  |  |  |
| - Non-respiratory infections (e.g. malaria, meningitis/encephalitis) |  |  |  |
| - Tuberculosis |  |  |  |
| - Malnutrition (e.g. SAM/MAM) |  |  |  |
| - Trauma / Injury |  |  |  |
| - Neurological conditions (e.g. seizures) |  |  |  |
| Adults |  |  |  |
| - All conditions |  |  |  |
| - COPD |  |  |  |
| - Tuberculosis |  |  |  |
| - HIV related infections |  |  |  |
| - Malignancy |  |  |  |
| - Non-respiratory infections (e.g. malaria) |  |  |  |
| - Pregnancy |  |  |  |
| - Trauma / Injury |  |  |  |
| - Neurological conditions (e.g. stroke, seizures) |  |  |  |
